# That Decision

**Published:** 1894-04

**Authors:** 


THAT DECISION.—What constitutes a legal practitioner in Texas? Our readers will recall the case of James Kennedy, M. D., vs. Schultze (San Antonio) for a surgical fee. Defendant put in the plea that Doctor Jas. Kennedy was not lawfully a practitioner, and showed that he had not received a license from the District Board—as the law requires—notwithstanding Dr. Kennedy has a diploma from the N. Y. College of Physicians and Surgeons, and is a professor in the Texas Medical College. Kennedy showed that there was no Board in existence in that District and pleaded that the law could not require a citizen to do an impossible thing. Nevertheless the court decided against him by judgment for defendant, and the doctor didn't get his fee. He appealed his case and the Court of Appeals has just sustained the decision. Dr. Kennedy will prepare a paper on the subject for our next number. This is a matter that vitally affects every physician practicing in Texas.

